# Immunoglobulin Replacement Therapy: Insights into Multiple Myeloma Management

**DOI:** 10.3390/cancers16183190

**Published:** 2024-09-18

**Authors:** Ilaria Saltarella, Concetta Altamura, Antonio Giovanni Solimando, Simona D’Amore, Roberto Ria, Angelo Vacca, Jean-François Desaphy, Maria Antonia Frassanito

**Affiliations:** 1 Section of Pharmacology, Department of Precision and Regenerative Medicine and Ionian Area (DIMEPRE-J), University of Bari Aldo Moro, 70124 Bari, Italy; ilaria.saltarella@uniba.it (I.S.); concetta.altamura@uniba.it (C.A.); jeanfrancois.desaphy@uniba.it (J.-F.D.); 2 Section of Internal Medicine and Clinical Oncology, Department of Precision and Regenerative Medicine and Ionian Area (DIMEPRE-J), University of Bari Aldo Moro, 70124 Bari, Italy; antonio.solimando@uniba.it (A.G.S.); simona.damore@uniba.it (S.D.); roberto.ria@uniba.it (R.R.); angelo.vacca@uniba.it (A.V.); 3 Section of Clinical Pathology, Department of Precision and Regenerative Medicine and Ionian Area (DIMEPRE-J), University of Bari Aldo Moro, 70124 Bari, Italy

**Keywords:** hypogammaglobulinemia, immunomodulation, immunoglobulin replacement therapy, multiple myeloma, pharmacology

## Abstract

**Simple Summary:**

Immunoglobulin (Ig) replacement therapy (IgRT) consists of the administration of low-doses human polyclonal Igs for the treatment of primary and secondary hypogammaglobulinemia, characterized by low serum levels of immunoglobulins that is associated with recurrent infections and immune dysfunction. In this review, we focus on the application and efficacy of therapeutic Igs for the management of multiple myeloma (MM) patients affected by secondary hypogammaglobulinemia that is associated with poor patients’ outcome. The use of IgRT restores physiological antibody levels and stimulates innate and adaptive immune responses as well. Therefore, in MM settings the IgRT has shown a significant positive impact on infection rates increasing the patients’ overall health status that correlates to a decrease in long-term complications and hospitalization and to an improved therapeutic adherence and patients’ quality of life.

**Abstract:**

Immunoglobulin (Ig) replacement therapy (IgRT) consists of the administration of low-dose human polyclonal Igs for the treatment of primary and secondary hypogammaglobulinemia that are associated with recurrent infections and immune dysfunction. IgRT restores physiological antibody levels and induces an immunomodulatory effect by strengthening immune effector cells, thus reducing infections. Here, we describe the pharmacology of different Ig formulations with a particular focus on their mechanism of action as low-dose IgRT, including the direct anti-microbial effect and the immunomodulatory function. In addition, we describe the use of therapeutic Igs for the management of multiple myeloma (MM), a hematologic malignancy characterized by severe secondary hypogammaglobulinemia associated with poor patient outcome. In MM settings, IgRT prevents life-threatening and recurrent infections showing promising results regarding patient survival and quality of life. Nevertheless, the clinical benefits of IgRT are still controversial. A deeper understanding of the immune-mediated effects of low-dose IgRT will provide the basis for novel combined therapeutic options and personalized therapy in MM and other conditions characterized by hypogammaglobulinemia.

## 1. Introduction

Immunoglobulins (Igs) are glycoproteins produced by plasma cells in response to antigenic stimuli in physiological and pathological processes. Igs are subdivided into five different classes, i.e., IgG, IgA, IgM, IgD, and IgE, which show different structures and functions [1,2]. IgG is the most abundant immunoglobulin (75–80% of serum Igs), followed by IgA, IgM, and the other Igs. In contrast, IgA is the most abundant isotype in mucosal secretions [3]. Physiologically, Igs are involved in many functions including host acute and long-term protection (IgM and IgG, respectively), defense against parasitic infections (IgE), mucosal protection (IgA), and activation of innate and adaptive immune cells via Ig fragment crystallizable (Fc) portion binding [4]. The level of circulating IgG ranges from 800 to 1800 mg/dL in healthy adults, while the levels of IgA are 90–400 mg/dL, IgM 60–280 mg/dL, IgD 0.3–0.4 mg/dL, and IgE 20–440 mg/dL [1]. These levels are influenced by age, sex and other factors (e.g., infections or inflammatory conditions) [5]. Different states may reduce circulating antibody levels, causing hypogammaglobulinemia, which has been defined by the European expert consensus as “serum IgG levels < 4 g/L” 6.

Hypogammaglobulinemia can be genetically determined or acquired. Primary hypogammaglobulinemias result from genetic disorders and/or chromosomal anomalies during the development of the immune system. These include Common Variable Immunodeficiency (CVID), X-linked agammaglobulinemia (XLA), and Severe Combined Immunodeficiency [6,7]. Conversely, secondary hypogammaglobulinemia is caused by other pathological conditions, such as hematological malignancies (e.g., multiple myeloma, lymphomas, chronic lymphocytic leukemia), transplantation, HIV infection, and/or pharmacological treatments with immunosuppressive drugs. Both conditions lead to several immune dysfunctions including recurrent infections, allergies, autoimmune diseases, and neoplasms [7]. Therefore, to prevent immunodeficiency-related comorbidity, hypogammaglobulinemia should be promptly treated. Besides antibiotic prophylaxis and vaccination, the management of hypogammaglobulinemia-affected patients includes immunoglobulin replacement therapy (IgRT), consisting of the administration of human polyclonal IgG to restore physiological IgG levels [8,9]. Usually, Igs are available as low-dose or high-dose formulations [10]. Low doses are indicated for the treatment of both primary and secondary immunodeficiencies [11]. In these diseases, Igs exert both a replacement activity and an immune-regulatory function through the stimulation of immune effector cell activity [10]. Interestingly, the immunomodulatory effect of therapeutic Igs is further enhanced at higher doses through the inhibition of dendritic cells (DCs), the expansion of T regulatory cells (Tregs), and the neutralization of autoantibodies [10,12]. High-dose formulations are currently indicated for the treatment of some neurologic and inflammatory disorders (e.g., myasthenia gravis, chronic inflammatory demyelinating polyneuropathy (CIDP), multifocal motor neuropathy) [13,14], and some autoimmune diseases (e.g., autoimmune encephalitis, autoimmune neutropenia, dermatomyositis, systemic lupus erythematosus) [15,16,17]. Hence, besides the passive replacement activity, Igs show many other immune-regulatory and anti-inflammatory activities, opening new perspectives towards novel combined therapeutic strategies and/or novel therapeutic indications.

Here, we describe the pharmacology of different Ig formulations with a particular focus on their mechanism of action as replacement therapy and their potential immune-mediated effects. Finally, we focus on the application and efficacy of therapeutic Igs for the management of multiple myeloma (MM) patients affected by secondary hypogammaglobulinemia.

## 2. Pharmacokinetics of Ig Formulations

Commercially available Ig preparations are purified from plasma collected from thousands of healthy donors. Hence, Ig formulations closely resemble the Ig repertoire of normal human plasma, mainly constituted by polyvalent IgG, followed by IgA, IgM and marginal levels of the other Igs [18]. Ig preparations are usually available in intravenous (IV), subcutaneous (SC), facilitated (f) and intramuscular (IM) formulations with different pharmacokinetic properties and side effects [18]. The choice among these routes depends on several factors, including patient conditions, pharmacokinetics, and pharmacoeconomic considerations [19].

### 2.1. Intravenous Immunoglobulins (IVIG)

IVIG was the first formulation approved as IgRT. IVIG allows the administration of large volumes of Ig preparations every 3–4 weeks. They are usually available as 5% (50 mg/dL) or 10% (100 mg/dL) liquid or as lyophilized preparations. IVIG may be administered as low (from 400 to 600 mg/kg) or high doses (from 1000 to 3000 mg/kg) based on the therapeutic indications [10,20].

After administration, Ig levels immediately rise, achieving maximum plasma concentration within 2 h. Next, plasma levels decrease during the following 7 days due to IgG distribution into the lymphatic system and/or extracellular fluid, and finally decline slowly because of renal catabolism [19,20].

Phase I clinical studies revealed that the pharmacokinetics of IVIG show considerable variability among different patient populations. In subjects with normal Ig levels, the mean half-life ranges from 1.3 to 1.9 days, whereas in bone marrow transplant recipients, Igs display an extended half-life (from 3.5 to 12.5 days) that rises to 56.5 days in patients with chronic lymphocytic leukemia or MM [20,21,22,23]. These data suggest that IVIG exhibit intra- and inter-population variability highlighting the need for individualized dosing and patient monitoring [20].

The direct infusion of IVIG administration can result in several adverse effects, including headache, cough, fatigue, infusion site reaction, nausea, urticaria, sinusitis, increased blood pressure, diarrhea, dizziness, noninfectious meningitis and lethargy. The most serious side effects are allergic reactions, anemia, breathing difficulties, swelling of the tongue or face, and skin rash [24].

Therefore, despite IVIG therapy being a powerful tool in the management of immune disorders and allowing rapid increase of circulating Ig, it requires assistance from nurses for administration, and the infusion will take several hours.

### 2.2. Subcutaneous Immunoglobulins (SCIG)

SCIG have a different pharmacokinetic profile compared to IVIG. SCIG are administered once or twice weekly in smaller doses (~100–200 mg/mL) than IVIG. Administration of SCIG involves the direct injection into the subcutaneous tissue that allows a slow release into the blood, reducing fluctuation of serum Ig levels that occurs with the IV route. Hence, the weekly administration of SCIG promotes physiological levels of Igs, providing a steady Ig level and ensuring the maintenance of their function [22,24,25,26,27].

A pharmacokinetic study showed that the SC administration of ^125^I-labeled anti-Rho Ig reaches the maximum plasma levels (~33% of the injected dose) after 4–6 days, and that serum half-life ranges between 22 to 46 days [27]. A more recent study showed a median half-life of 40.6 days for total IgG and of 23.3 days for tetanus antibodies, suggesting that endogenous IgG production may slow the elimination of total IgG [28].

Furthermore, SCIG therapy offers potential advantages in terms of administration and patient quality of life. SCIG is self-administered at home, providing greater convenience and flexibility for patients [29]. Nevertheless, the need to inject large volumes of Igs and the limited capacity of recipient tissue requires frequent administration at multiple sites. Additionally, mechanical devices, such as infusion pumps, that allow the SC administration may be difficult to use for some patients and may increase the total cost of the treatment regimen [29]. These features represent some of the major limitations of SC administration and are unfavorable for patients. Overall, SCIG are well tolerated, with fewer systemic adverse effects compared to IVIG owing to the low fluctuation of Ig levels [24,29]. Side effects are usually mild and include local reactions at the injection site, such as redness, swelling, and itching [24,29].

Therefore, SCIG are an effective option for the treatment of different pathological conditions, including primary immunodeficiencies (PID), chronic inflammatory demyelinating polyneuropathy (CIDP), and secondary hypogammaglobulinemia [30,31,32]. In addition, long-term SCIG therapy maintains functional stability with fewer fluctuations in symptoms compared to IVIG, highlighting their great potential in long-term patient management [22].

Thanks to the favorable pharmacokinetic profile, clinical efficacy, and advantages of SC administration, SCIG are a promising alternative to IVIG for the treatment of immunodeficiencies.

### 2.3. Facilitated Subcutaneous Immunoglobulins (fSCIG)

fSCIG are a form of Ig therapy in which recombinant human hyaluronidase PH20 (rHuPH20) is co-administered with Igs. rHuPH20 is an enzyme that degrades hyaluronan in the subcutaneous tissue, increasing its permeability and Ig absorption and dispersion [33].

The pharmacokinetics of fSCIG differs from those of regular SCIG since the presence of rHuPH20 facilitates the absorption of larger Ig volumes, allowing less frequent infusions compared to traditional SCIG. The co-administration of rHuPH20 results in a rapid increase in serum IgG levels that reach the plateau phase within the first 15 min of infusion. In addition, fSCIG have a bioavailability of 93% implying that a great amount of the administered dose reaches the systemic circulation, leading to improved therapeutic outcomes [34]. Body mass and age may influence the pharmacokinetics of fSCIG. Results from a phase III clinical trial showed that fSCIG induces fewer systemic side effects compared to IVIG (NCT03054181, [35]). Severe reactions were uncommon and included infusion site pain, swelling and genital edema. Antibodies to rHuPH20 were detected in 18% of patients included in the trial. Nevertheless, these antibodies were non-neutralizing and showed no correlation with adverse effects [36].

In conclusion, the pharmacokinetics of fSCIG offer several advantages over traditional SCIG, including higher bioavailability, faster absorption, and the ability to administer larger volumes during each infusion.

### 2.4. Intramuscular Immunoglobulins (IMIG)

The IM injection allows Ig administration into the muscle tissue with faster absorption and a longer half-life compared to SCIG. IMIG administration determines a depot at the injection site that results in a slow and prolonged release of the Igs [37]. One pharmacokinetic study using ^125^I-labeled anti-Rho Igs showed that IMIG induces a rapid uptake that reaches the maximum plasma level within 2–4 days, corresponding to ~40% of the injected dose [27]. Due to the limited capacity of the muscle tissue, IMIG formulations may be administered in small volumes (~3–5 mL) compared to IVIG and SCIG [38]. Thus, IMIG is uncommonly indicated for the treatment of immunodeficiencies, but it can be used as single-dose treatment for short-term prevention of infectious diseases (e.g., hepatitis A or tetanus prophylaxis) [39]. Despite IMIG effectiveness they are less commonly used compared to IVIG or SCIG since IM injections are painful and can cause local muscle damage with an increase in creatine kinase levels [40]. Other complications of the IM route of administration are abscess, indurations, bleeding and hematoma that in immunocompromised patients may often lead to local skin and soft tissue infections, including *S. aureus* [41].

## 3. Immune-Mediated Effect of IgRT

Low-dose Igs are mainly indicated as IgRT, acting as a resource of polyclonal pathogen-specific antibodies [42]. As Igs are usually purified from the human serum of thousands of healthy subjects exposed to pathogens and/or vaccines, the commercially available Igs contain a pool of antibodies able to prevent bacterial and viral infections [43]. Like physiological antibodies, therapeutic Igs exert multiple anti-microbial and immunomodulatory effects via the engagement of the dimeric antigen-binding fragment F(ab′) and of the Fc portion [44]. The F(ab′) fragment acts for antigen recognition, ensuring antigen neutralization and inactivation. On the other side, the Fc portion modulates immune response, acting as a bridge between the adaptive and innate immunity [44]. The binding of Fc to Fc Receptors (FcR) expressed on immune effector cells leads to antigen and target cell depletion via other Fc-dependent mechanisms, namely the antibody-dependent cell-mediated cytotoxicity (ADCC) and the antibody-dependent cellular phagocytosis (ADCP) [45]. In ADCC, Fc/FcR engagement induces the lysis of target cells via the release of perforins and granzymes by NK cells and other effector cells (e.g., macrophages, DCs, neutrophils, and eosinophils) [46]. In ADCP, the antibody induces antigen opsonization that promotes pathogen phagocytosis by macrophages [47]. Additionally, the binding of the Fc fragment to C1q activates the complement cascade that generates the membrane attack complex (MAC), a transmembrane channel that causes the osmotic lysis of the target cell [48]. The activation of the complement cascade also produces active opsonin C3b that binds to pathogen surfaces, further promoting target phagocytosis [48]. Overall, these mechanisms are essential for the induction of an effective immune response against microorganisms and pathogens that prevents recurrent infections [42,44].

In addition, low-dose Igs exert an immunomodulatory effect by inducing the activation and expansion of effector cells including B and T cells [42]. Based on these properties, Igs “correct” defective immune signaling in patients with immunodeficiencies that involve B cells, hence the antibody production, and other adaptive and innate immune cells as well. Indeed, patients eligible for IgRT exhibit reduced memory CD27^+^IgM^−^IgD^−^ B cells, impaired B cell differentiation, loss of CD4^+^ naive T cells [49], unbalanced CD4:CD8 T cell ratio [50], and defective DCs differentiation, maturation and function [51].

IgRT actively stimulates other B cell functions. Bayry et al. [51] showed that low-dose IVIG induces de novo IgM and IgG production and activates MAPK pathways, triggering B cell proliferation in CVID patients. The activation of B cells was associated with a decrease of inflammatory cytokines (e.g., IFN-γ, IL-12p70, IL-6), suggesting that IVIG reduces the inflammatory responses and the Th1 polarization associated with autoimmunity [51].

DCs act as professional antigen-presenting cells functioning as a bridge between innate and adaptive immunity. In vitro treatment of DCs from XLA and CVID patients with low-dose Igs induces the expression of CD1a, a DC maturation marker, and of co-stimulatory molecules, e.g., CD80, CD86, CD40, and HLA-DR that are essential in DCs-T cells cross talk [52,53]. The improved DC maturation is driven by IL-10 release, a reduction of IL-12, and by the activation of the CREB-1 pathway, suggesting that IgRT enhances DC maturation and T cell activation without inducing Th1 differentiation [53].

Regarding the T cell compartment, IgRT enhances CD4^+^ T cells and reduces the expression of activation (Ki67 and HLA-DR) and exhaustion (PD-1, CTLA-4) markers [12,54,55]. Following IVIG initiation, the amount of CD4 cells increases in the majority of CVID patients reaching normal circulating levels that may remain stable up to 1 year after the first IVIG treatment. These data suggest the ability of low-dose Igs to prevent aberrant T cell activation and to restore healthy T cell function, avoiding the inflammatory status of patients with hypogammaglobulinemia [55].

Recently, Simon-Fuentes et al. [56] performed a transcriptional analysis on peripheral blood mononuclear cells (PBMCs), CD14^+^ monocytes, CD3^+^ T cells, and CD20^+^ B cells from CVID patients treated with low-dose IVIG. Gene ontology analysis performed six hours after the infusion revealed prompt acquisition of an anti-inflammatory and immunosuppressive gene profile in PBMCs and CD14^+^ monocytes. Interestingly, both flow cytometry and gene expression analysis showed that IVIG modulates the balance among monocyte phenotypes (e.g., classical, intermediate, and non-classical) with an increase of the classical monocyte subset and a significant reduction of TNF expression. In line with these results, IVIG enhances the immunosuppressive CD11b^+^CD14^+^CD15^−^HLA-DR^− /low^ monocytic (m) myeloid-derived suppressor cells (MDSCs) (m-MDSCs) that suppress CD4^+^ T cell proliferation in m-MDSCs:CD4^+^ T cells co-cultures. These data highlight that IVIG-induced immunomodulation is mediated by the acquisition of an m-MDSC-like phenotype that might further emphasize IVIG immunoregulatory effects [56].

**Figure 1:**
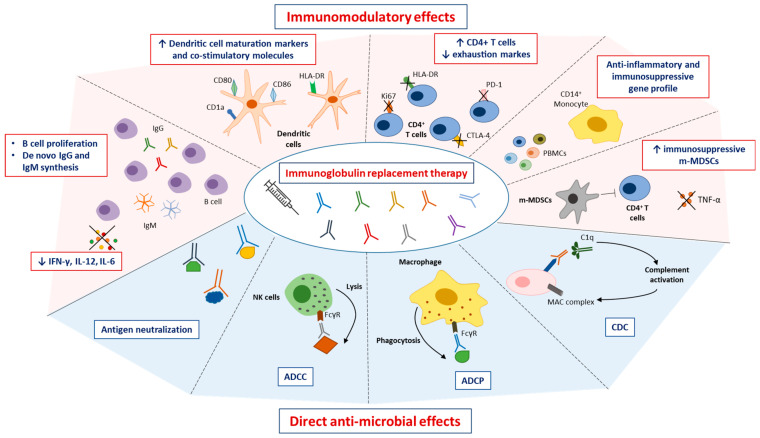
Mechanism of action of IgRT. Low-dose IgRT exerts multiple anti-microbial and immunomodulatory effects via the engagement of the dimeric antigen-binding fragment F(ab′) and of the fragment crystallizable (Fc) portion. The Igs induce direct anti-microbial effects via: (i) antigen neutralization; (ii) the antibody-dependent cell-mediated cytotoxicity (ADCC); (iii) the antibody-dependent cellular phagocytosis (ADCP); and (iv) the complement-dependent cytotoxicity (CDC). Igs have immunomodulatory effects by acting on different immune effector cells, including B cells, T cells, dendritic cells (DCs), peripheral blood mononuclear cells (PBMCs), CD14^+^ monocytes and monocytic myeloid-derived suppressor cells (m-MDSCs). ↑: increase; ↓: reduction. Figure adapted from Saltarella et al. [57].

Overall, these studies demonstrate that IgRT in primary and secondary hypogammaglobulinemia does not merely represent a passive transfer of antibodies to prevent recurrent infections but also plays an active role in regulating the immune response through the modulation of innate and adaptive immune cell activity. The mechanisms of action of therapeutic Igs are illustrated in Figure 1.

## 4. Multiple Myeloma (MM) and Secondary Hypogammaglobulinemia

MM is a hematologic cancer characterized by clonal proliferation of tumor plasma cells that produce high levels of monoclonal Igs, also known as paraprotein [57,58]. MM typically evolves from the premalignant state of monoclonal gammopathy of undetermined significance (MGUS) to Smoldering Myeloma (SMM) that over time develops into clinically overt MM due to acquired genetic mutations and alterations of the surrounding microenvironment [59].

MM cell growth in the bone marrow hampers healthy plasma cells that gradually decrease, resulting in the accumulation of monoclonal Igs and a reduction of circulating polyclonal antibodies, giving hypogammaglobulinemia [59,60,61]. Hypogammaglobulinemia is diagnosed by assessing the total concentrations of IgG, IgA, and IgM. Nevertheless, Ig paraprotein produced by MM cells, as well as therapeutic moAbs, may interfere with the measurement of IgG levels, hindering hypogammaglobulinemia diagnosis. For these reasons, hypogammaglobulinemia in MM patients is often underestimated, and recent recommendations for management of secondary antibody deficiency in MM state that a serum IgG concentration < 4 g/L should be defined as “severe hypogammaglobulinemia”, and that serum IgG concentrations between 4 and 6 g/L should be defined as “mild hypogammaglobulinemia” [62]. To support hypogammaglobulinemia diagnosis, alternative methods that exclude paraproteins and therapeutic moAbs have been developed, including the Calculated Globulin screening test [63] and the Antigen Specific therapeutic monoclonal Antibody Depletion Assay (ASADA) [64]. Despite these methods allowing an accurate assessment of IgG levels, they are not validated and are not routinely employed in clinical practice. Furthermore, the evaluation of functional CD19^+^ B cells may provide insights on residual humoral immunity of MM patients [62].

Hypogammaglobulinemia is a common feature of MM patients that occurs in up to 90% of patients. Giralt et al. showed that the premalignant phases of MGUS and SMM have a greater risk of developing hypogammaglobulinemia, suggesting the existence of immune dysfunction already in the early stages of the disease [62]. Accordingly, several studies have documented that MGUS to MM transition is driven by immune dysfunction. Indeed, cytokines and immune-regulatory pathways sustain cancer immunoediting by creating an immunosuppressive environment that supports MM cell survival, angiogenesis and immune evasion [65,66,67]. Transcriptomic analysis of the bone marrow microenvironment showed T cell polarization towards an exhausted phenotype and an increase of immune cells with immunosuppressive function [68,69,70]. Zelle-Rieser et al. demonstrated that MM patients’ T cells express inhibitory molecules (PD-1, CTLA-4, CD160) and senescence cell markers (expression of CD57 and loss of CD28) [70]. Similarly, NK cells are dysfunctional with an aberrant expression of inhibitory checkpoints (KIR and NKG2A) and activation markers (CD137 and CD69) [71,72,73]. In addition, high levels of TGFβ, IL-6 and IL-1β in the bone marrow niche promote T_H_17 polarization, contributing to immune evasion and osteolytic lesions [74,75,76], and sustain the expansion of CD4^+^CD25^high^FoxP3^+^ Tregs that suppress the anti-tumor response, hence supporting MM cell survival [77].

Overall, the decrease of healthy plasma cells, as well as the immunosuppressive environment, causes an increased infection rate that negatively affects patient outcome. Several studies showed that infections are the major cause of death in MM patients [62]. Retrospective studies showed that bacterial infections were responsible for ~50% of hospitalized MM patients’ deaths, and that the mortality rate correlated with markers of disease activity including plasmocytosis, C-reactive protein and β-2 microglobulin [78]. A retrospective study of over 90 newly diagnosed MM patients who developed sepsis found that gram-negative and gram-positive bacteria, as well as fungi, were common causative pathogens. Nearly a quarter of cases were attributed to each of these classes of microbes [79]. Furthermore, even among the 18% of culture-negative sepsis patients, clinical indicators like elevated SIRS (Systemic Inflammatory Response Syndrome) scores supported the infectious etiology. This highlights the challenges of detecting causative agents and the importance of considering sepsis clinically. Certain patient factors correlated with poorer progression-free survival outcomes, including albumin levels < 3.5 g/dL, lower Karnofsky performance status, more advanced disease stage per standardized rating scales, hypogammaglobulinemia, and immunoparesis [80]. The incidence and impact of sepsis emphasize MM patients’ vulnerability to opportunistic infections. Preventive approaches and optimized treatment based on stratifying infection risk may help mitigate this challenge and improve clinical management for those with newly diagnosed disease [79,81].

Main clinical manifestations of hypogammaglobulinemia involve the respiratory tract (e.g., pneumonia, bronchitis, bronchiolitis), otitis or urinary infections, chronic diarrhea due to gastrointestinal immunodeficiency, and consequent bacterial, viral, and fungal proliferation, enlarged lymph nodes, and/or spleen [82]. The frequent occurrence of acute and severe infections negatively affects the quality of life of patients decreasing their social participation and increasing healthcare costs [83,84,85].

Hypogammaglobulinemia is exacerbated by MM progression and pharmacological treatments, including those with monoclonal antibodies (mAbs, e.g., daratumumab, belantamab mafodotin, bi-specific antibodies) and corticosteroids [86,87,88,89]. Several clinical studies have shown that MM patients treated with the anti-CD38 daratumumab, as well as anti-BCMA bi-specific antibodies, may develop severe hypogammaglobulinemia [85,86,87].

Overall, the development of secondary hypogammaglobulinemia in MM patients points to the intricate relationship between MM cells and the immune system, providing the rationale for innovative treatment strategies that simultaneously target tumor cells and enhance immune effector functions.

## 5. Clinical Evidence of IgRT Efficacy in MM

Low-dose IgRT has acquired increasing importance for the management of secondary hypogammaglobulinemia in MM [6,62].

In 1994, a multicenter randomized clinical trial that enrolled 82 MM patients treated with IVIG (0.4 g/kg) or with placebo (0.4% albumin) for 1 year first showed the effectiveness of IgRT in MM. Nineteen IVIG-treated patients developed serious infections versus 38 patients treated with placebo, thus demonstrating the efficacy of IgRT in reducing the risk of severe and recurrent infections in MM patients [90]. Other clinical trials confirmed the ability of IgRT to prevent life-threatening and recurrent viral, bacterial and mycotic infections, showing reduced infection rates, reduced use of antibiotics and hospitalization periods, and improvement of patient survival and quality of life [89,91]. MM patients receiving IgRT display improved patient satisfaction [90,91]. By contrast, a retrospective study including 266 MM patients undergoing autologous stem cell transplantation (ASCT) between 2000 and 2009 showed that Ig replacement therapy did not reduce the occurrence of infective episodes, including bloodstream infections, pneumonia, gastrointestinal or urinary tract infections [92]. Girmenia et al. [93] published expert panel consensus-based recommendations after a meeting held in 2017. The authors established that the use of IVIG is not recommended routinely for patients with MM, and it may be reserved for patients with very low IgG levels (<400 mg/dL) and recurrent life-threatening infections [93]. More recently, a European expert consensus for the treatment of secondary antibody deficiency states that IgRT represents an important therapeutic option for patients with hematological malignancy and hypogammaglobulinemia (IgG levels < 4 g/L) who experience severe, recurrent or persistent infections despite anti-infective treatment [6]. In MM settings, Giralt et al. suggest the use of IgRT for infection prophylaxis in patients with serum IgG concentrations > 6 g/L but with recurrent infections and a poor vaccine response. Furthermore, the authors proposed a management algorithm for IgRT initiation and discontinuation that may guide physician decision-making to improve patient management [62].

The introduction of SCIG formulation, which can be self-administered at home, has further improved patients’ quality of life with advanced compliance and therapeutic adherence. Vacca et al. investigated the efficacy of SCIG in MM patients with secondary hypogammaglobulinemia. Patients treated with SCIG showed a significantly lower rate of serious bacterial infections compared to untreated patients, resulting in effective reduction of the annual rate of severe infections. In addition, SCIG therapy significantly reduced the frequency of illness episodes, alleviated fatigue, halted the burden of hospital visits and recovery for infectious complications, and reduced the use of antibiotics. Therefore, the lower infection risk contributes to improved emotional and psychological patient condition with a positive impact on the overall quality of life evaluated through the SF-36 questionnaire [94].

Overall, the European Medicines Agency recommended both IVIG and SCIG low-dose formulations for patients with severe or recurrent infections who are non-responders to anti-microbial treatments and have low serum IgG levels [95,96]. The choice between IVIG and SCIG should consider the advantages and disadvantages of both routes of administration including frequency of administration, adverse events, and self- versus nurse-administration [62].

Despite the advent of mAbs (e.g., the anti-CD38 mAbs, bi-specific antibodies) having enhanced the overall survival of MM patients [97], their use has been associated with a higher risk of developing hypogammaglobulinemia and severe infections [98]. The increased infection rate of MM patients undergoing anti-CD38 therapies is due to the CD38 expression on both malignant and healthy plasma cells and on other immune effector cells (e.g., NK cells), which leads to a reduction of polyclonal Igs and innate immune cell response [57,99]. Accordingly, patients receiving anti-CD38-based therapy have an increased risk of developing infections compared to patients treated with other backbone MM regimens [98]. The most severe infections of daratumumab-treated patients involve the respiratory tract [98]. A retrospective study of MM patients treated with daratumumab in combination with other anti-MM regimens (Immunomodulatory drugs—IMiDs-, proteasome inhibitors, cytotoxic chemotherapy, or panobinostat) documented that IVIG IgRT during daratumumab-based therapy significantly reduced the total infection rate (39%), resulting in a reduction of 72% of severe grade 3–4 infectious episodes [100]. As IVIG therapy did not correlate with other adverse reactions or complications, the early introduction of IVIG may represent a useful and safe strategy for patients undergoing daratumumab-based combinations [101,102,103].

Furthermore, a retrospective study including MM patients receiving bi-specific antibody therapies (e.g., BCMA, GPRC5D and FCRH5) showed a correlation with all-grade infections (13–76%). Interestingly, this study highlights that viral infections were the most common, including rhinovirus/enterovirus, adenovirus, SARS-CoV-2, and influenza suggesting the need to improve prevention strategies against respiratory viruses. Virus reactivation (e.g., cytomegalovirus) was responsible for 7% of microbiologically determined infections in MM patients undergoing bi-specific antibody therapies. Among these, the widely used T cell/BCMA bi-specific antibody resulted in the most correlated with all-grade infections [104]. Accordingly, the anti-BCMA bi-specific antibody therapy has been associated with severe cytopenia and grade 3–5 infections, including some fatal ones due to COVID-19 and/or other pathogens [105]. IVIG replacement therapy correlated with a decrease of 90% in grade 3–5 infections compared with periods ‘Off-IVIG’, suggesting a role for IVIG as primary prophylaxis in preventing serious infections in patients treated with the BCMA-targeting bi-specific antibody [105]. No significant differences were observed in grade 1–3 infections [105]. Lim et al. confirmed the efficacy of IVIG during therapies with daratumumab and anti-BCMA bi-specific antibodies in reducing by 40% the rate of all-grade infections per year, providing important findings for the application of IgRT for MM patients in combination with novel immunotherapies including bi-specific antibodies [98,104]. Hence, monitoring IgG levels and immune subsets is currently recommended for patients treated with anti-BCMA bi-specific antibodies eligible for prophylactic IgRT [62].

Interestingly, IgRT may have potential effectiveness in the management of hypogammaglobulinemia induced by CAR-T cell therapy in hematological malignancies. Hypogammaglobulinemia occurs in 94% of MM patients undergoing CAR-T cell therapy and was associated with increased risk of infections, especially respiratory ones, induced by bacteria in the early period and by viruses in the following months [106,107]. To date, no randomized clinical studies evaluating the efficacy of IgRT in CAR T cell recipient patients have been conducted. Nevertheless, Hill et al. [108] proposed an algorithm that recommends IgRT in patients with IgG ≤ 400 mg/dL and persistent and recurrent infections in the first 3 months after receiving anti-CD19 CAR-T cell therapy. Therefore, despite the limited evidence supporting prophylactic IgG replacement based on IgRT safety profile and reduced regimen costs, it should be considered for patients undergoing CAR T cell therapy [109].

In conclusion, IgRT has a significant positive impact on infection rates, increasing patients’ overall health status that correlates to a decrease in long-term complications and hospitalization and to improved therapeutic adherence and patients’ quality of life.

## 6. Conclusions

IgRT allows the administration of human polyclonal therapeutic Igs as a key strategy for the management of patients with primary and secondary hypogammaglobulinemia. The use of IgRT restores physiological antibody levels and stimulates innate and adaptive immune responses as well. Besides their passive replacement activity, Igs have immune-regulatory and anti-inflammatory effects, which are further enhanced at higher doses. The use of high-dose Ig formulations for the treatment of autoimmune diseases and inflammatory diseases suggests that the therapeutic potential of Igs may extend beyond their traditional use in immunodeficiency disorders. Overall, IgRT improves patients’ quality of life by preventing immunodeficiency-related comorbidities, including life-threatening infections.

Hypogammaglobulinemia is a common condition in MM further sustained by disease progression and pharmacological treatments, resulting in a higher susceptibility to infections that negatively affects patient outcome and overall survival. In MM settings, data on IgRT dosing are limited, with most evidence supporting low-dose therapy for reducing infections. High-dose IgRT remains understudied, so low-dose is the current standard, tailored to patient needs. More research on high-dose IgRT is needed for clearer guidance. Without better recording and reporting of immunoglobulin levels in these patients, the effectiveness of key management strategies—such as infection prevention, vaccination, antibiotics, antivirals, and IgRT—cannot be fully assessed or optimized [110]. Overall, low-dose IgRT enhances the anti-microbial immune system by restoring circulating antibody levels, thereby reducing the risk of infections. However, the clinical benefits of IgRT are controversial. These controversial data may be due to the heterogeneous MM therapies based on the combination of two or more drugs with immunosuppressive effects that further affect hypogammaglobulinemia, concealing the efficacy of therapeutic Igs, and/or to the lack of large and homogeneous studies based on personal clinicians’ experience.

Future studies investigating the immune-regulatory and anti-inflammatory effects of high- and low-dose Igs could potentially demonstrate the activation of the immune system in MM patients. Hence, the immuno-mediated role of IgRT in MM should represent a promising field of research, opening new perspectives towards a deeper understanding of immune system response and providing the basis for novel combined therapeutic options. The introduction of personalized therapeutic approaches with constant monitoring of patients undergoing IgRT through the evaluation of IgG levels, immune system activation, as well as infection rates should improve patients’ management and outcome.
